# Distribution of Kell phenotype among pregnant women in Sokoto, North Western Nigeria

**DOI:** 10.11604/pamj.2015.21.301.4636

**Published:** 2015-08-26

**Authors:** Erhabor Osaro, Malami Aisha Ladan, Isaac Zama, Yakubu Ahmed, Hassan Mairo

**Affiliations:** 1 Department of Haematology and Transfusion Science, Faculty of Medical Laboratory Science, Usmanu Danfodiyo University, Sokoto, Nigeria; 2 Department of Obstetrics and Gynaecology Usmanu Danfodiyo University, Sokoto, Nigeria

**Keywords:** Kell phenotype, pregnant women, Nigeria

## Abstract

**Introduction:**

Kell antigen is highly immunogenic and is the common cause of antibody production in mismatched blood transfusions, haemolytic transfusion reaction (HTR) and maternal alloimmunization, which causes severe anaemia in neonates. The aim of this study is to determine the prevalence and ethnic variation of the Kell phenotype among pregnant women in Sokoto, Nigeria.

**Methods:**

Kell antigen status of 150 pregnant women aged 18-45 years and mean age 27.19 ±4.69 years attending antenatal clinic in UDUTH Sokoto Nigeria was determined using the conventional tube method and anti-Kell reagents (Lorne Laboratories, UK).

**Results:**

Among the 150 subjects studied, 3 (2.0%) of subjects were positive and 147 (98.0) were negative for K antigen. Of the 150 pregnant subjects; 32 (21.3%) were primigravidae while 118 (78.7%) were multigravidae. Kell phenotype was more prevalent among primigravidae (3.1%) compared to multigravidae (1.7%) women. The distribution of Kell phenotype among the pregnant subjects was compared based on ethnicity. The prevalence of Kell antigen was significantly higher among the Hausa ethnic group (3.2%) compared to other ethnic groups which indicated zero prevalence (p = 0.001). Kell negative phenotype was ≥ 96.8% among all the ethnic groups.

**Conclusion:**

Our observed prevalence of Kell phenotype is consistent with previous studies among Blacks and Asians but significantly lower than values observed in previous studies among Caucasians. We recommend that all pregnant women should be screened for the presence clinically significant red cell antigens including Kell antigen on their first antenatal visit. Kell negative red cell should be routinely provided for all pregnant women and women with child bearing potential to reduce the risk of Kell-associated HDFN. There is need to introduce routine screening of pregnant women for clinically significant red cell antibodies to facilitate the effective management of HDFN as well as prevent HTR. There is also need for sustained health education of pregnant women in the area to encourage early booking for antenatal care.

## Introduction

The Kell blood group system is complex and has many antigens which are highly immunogenic. These antigens particularly KEL 1 are second to Rhesus D as a cause of Haemolytic Disease of the Foetus and New-born (HDFN) [1]. Antibodies that target Kell antigens can cause HDFN, as well as Haemolytic Transfusion Reactions (HTR) [2]. Anti-Kell related HDFN causes severe foetal anaemia as maternal anti-Kell target foetal RBC precursors, suppressing the foetal production of RBCs [3, 4]. The Kell blood group system was discovered in 1946 and was named after Mrs. Kelleher, a patient who had anti-Kell antibodies and whose baby had HDFN. The Kell blood group system is important in transfusion medicine and HDFN and is considered to be one of the major human blood group antigens. Antibodies produced against Kell antigens are predominantly IgG and can cross the placental barrier, does not bind complement and therefore causes is extravascular haemolysis. KEL is highly polymorphic, has two major codominant alleles, k and K, which result from a SNP (698C→T) and the corresponding k and K antigens differ by a single amino acid change [4–6]. Individuals without Kell antigens (K_0_) who have formed an antibody to a K antigen must be transfused with blood from donors who are also K_0_ to prevent severe haemolytic transfusion reaction [2, 7]. Kell isoimmunisation is the third most common cause of HDFN after Rh and ABO. Anti-Kell causes severe foetal anaemia by suppressing foetal RBC synthesis [8]. Anti-Kell is an important cause of HDFN and tends to occur in mothers who have had several Kell incompatible blood transfusions in the past and in mothers who have been sensitized to the Kell antigen during previous pregnancies. HDFN -associated anaemia is caused by the ability of anti-K to cause the suppression of foetal production of RBCs [9]. Unlike Rh and ABO, Kell antigens are expressed on the surface of RBC precursors, and anti-K promotes the immune destruction of K+ erythroid early progenitor cells by macrophages in the foetal liver rather than only mature foetal RBCs [10]. The RBC precursors do not contain haemoglobin and thus less bilirubin is released during the haemolysis, and jaundice in the new-born period is less common in Kell-related HDFN [11]. There is paucity of data on the prevalence of Kell phenotype among pregnant women in Sokoto, Nigeria. The risk of anti- Kell- related HDFN in Sokoto is unknown. Therefore the present study is aimed at determining the prevalence and ethnic variations of the Kell phenotype among pregnant women in Sokoto, Nigeria. Data generated will help improve the obstetrics care offered to pregnant women in the area and may help justify the need to routinely screen pregnant women in the area for clinically significant red cell antigens. Findings from this study will also help blood bank in the area to stock optimum levels of Kell negative units for use among pregnant women and women who are of child- bearing age and are Kell antigen negative as well as those who are positive for anti-Kell antibodies that require antigen negative red cell for transfusion.

## Methods

### Study area

***Study site:*** the selected area for this study is Usmanu Danfodiyo University Teaching Hospital (UDUTH) which is located in Wamakko Local Government within Sokoto Metropolitan city in Sokoto State. Sokoto State is located in the extreme Northwest of Nigeria, near the confluence of the Sokoto River and Rima River. With an annual average temperature of 28.30c (82.9 0F). Sokoto is, on the whole, a very hot area. However, maximum day time temperatures are for most of the year generally under 40 0C (104.0 0F). The warmest months are February to April when daytime temperatures can exceed 45 0C (113.0 0F). The rainy season is from May to October during which showers are a daily occurrence. There are two major seasons, wet and dry which are distinct and are characterized by high and low malarial transmission respectively. Report from the 2007 National Population Commission indicated that the State had a population of 3.6 million [12].

**Study subjects and design:** this case study included 150 consecutively- recruited pregnant women vising the antenatal clinic in Usmanu Danfodiyo Hospital Sokoto. Subjects were aged 18-45 years with mean age of 27.19 ±4.69 years. Blood samples were analysed for their Kell antigen status. Verbal informed consent was obtained from all study subjects. The study was approved by the ethical committee of Usmanu Danfodiyo University Teaching Hospital, Sokoto, Nigeria.

**Inclusion criteria:** all consecutively-recruited, consenting and confirmed pregnant (by a consultant obstetrician) women aged 15-45 years visiting the antenatal clinic of Usmanu Danfodiyo University Teaching Hospital (UDUTH) Sokoto were included in this study.

**Exclusion criteria:** the following were excluded from this study; non-pregnant women, non-consenting pregnant women, women who have had a red cell transfusion in the last 4 months and women who have had a recent stem cell or bone marrow transplant.

**Study site and participating hospital:** study will be conducted in the service laboratory of Usmanu Danfodiyo University Teaching Hospital (UDUTH) Sokoto, Nigeria. The hospital is a tertiary health facility rendering quality healthcare services to the people of Sokoto State and the neighbouring states of Zamfara and Kebbi State.

**Sample collection and testing:** three millilitres of blood sample was drawn aseptically with disposable plastic syringe from the median antecubital vein for all the subjects into dipotassium ethylenediamine tetracetic acid (K_2_EDTA) blood containers. 2-3% suspension of washed red cells from each study subjects was prepared in low ionic strength solution. Lorne Laboratories (UK) anti-Kell reagents were reacted with red cell suspension from each subject. The Lorne anti-Kell reagent will cause direct agglutination (clumping) of cells that carry K antigen. No agglutination generally indicates absence of K antigen.

## Results

In this study, the Kell phenotype of 150 pregnant women aged 18-45 years and mean age 27.19 ±4.69 years attending antenatal clinic in UDUTH Sokoto Nigeria was determined. Among the 150 subjects 3(2.0%) of subjects were positive for K and 147(98.0) were negative for K antigen. Table 1 shows the prevalence of Kell antigen among subjects.

**Table 1:** Prevalence of Kell antigen among subjects

Antigen	Number Tested	Number Kell positive	% Kell positive	Number Kell negative	% Kell negative
Kell	150	3	2.0	138	98.0

### Distribution of Kell antigen based on parity

Of the 150 subjects 32 were primigravidae (%) while 118 were multigravidae. Of the 32 primigravidae, 1 woman (3.1%) was Kell positive while 31 (96.9) were Kell negative. Also 2 out of the 118 multigravidae women (1.7%) were Kell positive while 116 (98.3%) were Kell negative. Table 2 show the distribution of K negative phenotype based on parity.

**Table 2:** Distribution of K negative phenotype based on parity

Parity	Number Tested	K Positive	% K Positive	K negative	% K egative	p-value
Primigravidae	32	1	3.1	31	96.9	0.001
Multigravidae	118	2	1.7	116	98.3	

### Distribution of subjects based on ethnicity

The distribution of Kell phenotype among the pregnant subjects was compared based on ethnicity. The Hausa ethnic group constituted the most predominant ethnic groups 94(62.7%) compared to 20(13.3%), Igbo 20(13.3%), Fulani 13(8.7%), others minority ethnic groups 13 (8.7%) and Yoruba 10(6.7%). The prevalence of Kell phenotype was significantly higher among the Hausa ethnic group (3.2%) compared to other ethnic groups that indicated zero prevalence (p = 0.001). Kell negative phenotype was ≥ 96.8% among all the ethnic groups.Table 3 shows the prevalence of Kell antigen among subjects based on ethnicity.

**Table 3:** Distribution of Kell phenotype based on ethnic group of subjects

Ethnic groups	Number Tested	Number K-positive	% Kell positive	Number K negative	% Kell negative	p-value
Hausa	94	3	3.2%	91	96.8%	0.001
Fulani	13	0	0	13	100%	
Yoruba	10	0	0	10	100%	
Igbo	20	0	0	20	100%	
Others	13	0	0	13	100%	

### Discussion

Information about the prevalence of blood group antigens in a population is useful for providing better blood transfusion services and in the effective and evidenced-based management of HDFN. This research work was carried out to determine the prevalence of Kell phenotype among pregnant women in Sokoto, North Western Nigeria. Among the pregnant women studied, we observed a Kell antigen prevalence of (2%). Our finding is consistent with previous report by Lamba and colleagues [13] who observed a Kell antigen prevalence of 2.8% among their cohort of 1,000 healthy and regular repeat blood donors in India. Our finding is also consistent with a previous report by Chaudhary and colleagues [14] among North Indian blood donors which reported a Kell prevalence of 1.92%. Similarly, Ugboma and Nwauche [15] in Port Harcourt investigated the prevalence of Kell antigen among their patients and reported a Kell antigen prevalence of 2%. Our observed prevalence of 2.0% is however lower than a prevalence of 5.56% observed by Thakral and colleagues [16] among North Indian blood donors. Our observed prevalence is also lower than that previously reported among Caucasians (9%) [17–19]. Our observed Kell prevalence is also lower than a 3.5% prevalence obtained by Makroo and colleagues [20] among their cohort of 3073 blood donors in India. Racial differences seem to exist in Kell blood group antigen distributions [13, 19]. A study of randomly selected 123 regular Maldivian blood group O blood donors who were phenotyped for Kell blood group antigen indicated a prevalence of 5.7% [21]. Similarly a previous report among 115 blood donors who were investigated in South Gujarat, India for extended antigen typing, reported no Kell phenotype [22]. Also, a previous study by Lin and colleagues [23] (1988) among Chinese subjects in Taiwan indicated a zero percent Kell prevalence. The prevalence of K phenotype among Senegalese is 0.5% [24]. A previous report on the prevalence of Kell blood group antigen in the Bengalee population observed a Kell phenotype prevalence of 0.8% [25]. Similarly, evaluation of Kell blood group antigen frequencies among Macedonian population by Makarovska-Bojadzieva and colleagues [26] indicated a Kell prevalence of 0.25%.

Kell blood group system is the next most clinically significant after the ABO and Rhesus blood group system [27]. Antibodies to the Kell antigen have been incriminated in HDFN and in haemolytic transfusion reactions [28–30]. Anti-K typically presents as IgG class alloantibody. Individuals lacking a specific Kell antigen may develop antibodies against Kell antigens when transfused with blood containing that antigen. This is particularly true for the K antigen which shows a relatively high antigenicity and moderately low frequency in African populations. The Kell antibodies low molecular weight IgG antibodies and can pass through the placenta barrier and cause HDFN in foetus positive for the Kell antigen [31]. The alloantibodies, which frequently develop and are encountered during compatibility testing, are primarily against antigens related to Kell blood group system [28]. Antibodies directed against Kell antigens are implicated in cases of HTRs and HDFN, and are, therefore, regarded as clinically significant particularly if these react in the indirect antiglobulin test at 37°C [28, 32, 33]. It is vital to routinely determine the frequencies of the various blood group antigens among blood donors population particularly with regards to meeting the red cell transfusion-related needs of patients who have developed multiple alloantibodies. This information is necessary to predict the availability of blood units that lack the corresponding antigen(s). A review of the prevalence of antibodies associated with HDFN in reproductive age women revealed a marked decrease in the incidence of anti-Rh D and an increasing incidence of anti-K antibody in the US and in other developed countries [34]. Anti-K was detected with a frequency of 1.0% in a previous study [29] that investigated 500 pregnant women for clinically significant red cell antibodies in Port Harcourt, Nigeria. Anti-K related HDFN can be severe and there is evidence that anti-K can recognize K antigens expressed in the early stage of erythroid development in the foetal liver and can cause anaemia by suppressing erythropoiesis [34, 35]. A previous study investigated the prevalence of red cell antibodies among pregnant women in Sweden and observed eight hundred and twenty-one alloantibodies in 629 immunized pregnant women with 753 foetuses. An overall antibody incidence of 0.57% was observed which included 373 clinically significant antibodies found in 261 mothers (0.24%). Multiple antibodies were present in 8.2% of all samples. Anti-K HDFN was reported in16 Kell-positive babies out of whom three required phototherapy and one required exchange transfusions [36].

Our finding underscores the importance of routine screening of pregnant women for clinically red cell antigens [37]. The phenotype of clinically significant red cell antigens on donor's red cells is important particularly if the blood is intended for transfusion to patients with alloantibodies to facilitate the transfusion of red cells that are negative for the antigens to which patient's antibodies are specific. Routine determination of clinically significant red cell antigens facilitates the planning and running of an efficient blood transfusion service and better management of HDFN. First-trimester screening of pregnant women enables timely identification and treatment of HDFN. Severe HDFN, caused by antibodies other than anti-D, is associated with anti-K, anti-c, and to a lesser extent with other Rh-alloantibodies [38]. The knowledge of clinically significant red cell antigens that are known to cause HDFN, HTR and shortened survival of red cell is important and can help in the prevention and appropriate management of HDFN as well as optimize transfusion among transfusion- dependent patients (sickle cell disease, thalassemia, haematological oncology, myelofibrosis myelodysplasia and aplastic anaemia) particularly those that have developed alloantibodies against clinically significant red cell antigens [39]. One strategy to decrease alloimmunization in transfusion dependent women of child bearing age is provision of phenotype matched RBCs for C, E, and K antigens [40]. Individuals lacking the Kell antigen may develop antibodies against Kell antigen when transfused with Kell positive red cells. These antibodies can potentially cause the destruction of Kell positive red cells in future transfusions. Similarly pregnant women who are Kell negative can be sensitized by feto maternal haemorrhage associated with pregnancy involving a Kell positive foetus [41].

Pregnant women with K antibodies will need to be effectively managed by a gynaecologist to reduce the risk of HDFN. Effective management will involve checking the paternal sample for their Kell antigen status. If the husband is Kell positive, there may be need to determine the foetal Kell genotype using maternal plasma at 20 weeks gestation [42]. If the foetal Kell genotype is positive, the anti-K titre will need to be monitored every 4 weeks until 28 weeks gestation and every 2 weeks thereafter. There is a high risk of HDFN if the anti-Kell titre is 32 or greater or if there has been a significant rise since last titration [43, 44]. At delivery it is important to monitor baby's blood group, direct antiglobulin test, haemoglobin and bilirubin [45]. Severely jaundiced baby may be managed with phototherapy or with exchange blood transfusion to prevent Kernicterus. The pathogenesis of HDFN caused by anti-K differs from anti-D. This is because anti-Kell associated HDFN is much harder to predict, as there is little association between anti-K titre and severity of the disease and anti-K associated HDFN is linked with lower concentrations of amniotic fluid bilirubin than in anti-D HDFN of corresponding severity. Also postnatal hyperbilirubinaemia may not present in babies with anaemia caused by anti-K. There is decreased reticulocytosis and erythroblastosis in the anti-K disease. Foetal anaemia in anti-K HDN is due to suppression of erythropoiesis. The Kell glycoprotein emerges on erythroid progenitors, by macrophages in the foetal liver, before they develop into haemoglobinized erythroblasts [46]. As RBC precursors contain no haemoglobin, the release of bilirubin is reduced during haemolysis, therefore jaundice is not as common, but severe anaemia may occur [3]. This is supported by in vitro studies which have shown that antibodies to Kell inhibit growth of KEL 1 progenitor cells. In order to prevent HDFN, it is necessary for the detection of the foetal KEL 1 gene [1]. In pregnant women with anti-KEL1 and who have a partner with KEL 1 and 2, prediction of KEL1 phenotype from tests on foetal DNA allows obstetricians to adjust management of the pregnancy based on whether the foetus is at risk of developing HDFN.

## Conclusion

Our study indicates that blood group antigens can be distributed differently within different nationalities. Our observed prevalence of Kell phenotype is consistent with previous studies among Blacks and Asians but significantly lower than values observed in previous studies among Caucasians.

### Recommendations

We recommend that all pregnant women should be screened for the presence clinically significant red cell antigens including Kell blood group antigens on their first antenatal visit. There is need to introduce routine screening of pregnant women in Sokoto Nigeria for clinically significant red cell antibodies to facilitate the management of HDFN as well as prevent HTR by enabling the selection of antigen negative red cells for women who have alloantibodies and require a red cell transfusion. Kell negative red cell should be routinely provided for all pregnant women with child bearing potential to reduce the risk of Kell-associated HDFN. There is also need for sustained health education of pregnant women in the area to encourage early booking for antenatal care and adherence to prescribed medication.

### Limitations

This study had some limitations. Firstly the sample size for this study was relatively small compared to the huge population of the North West zone in particular and the Nigeria nation in general. The prevalence obtained in this study gives an estimate on the frequencies of the Kell phenotype among pregnant women in Sokoto, North West, Nigeria. Cost implications as well as access to reagents was a limiting factor that affected the number of subjects included in this study. There is need for a nationwide study to determine the prevalence of Kell blood group antigens among Nigerians. Secondly, we used the conventional manual tube method in the screening of our cohort of pregnant women for Kell phenotype. Some previous reports which utilized more sensitive automated testing instrument using commercial antisera and gel card methods. Evidenced -based best practices in most developed countries indicates that the gel and glass beads-based card method has several advantages over the conventional tube method; paediatric friendly because they require small volume of patient specimen, unlike with tube methods, no washing of the antiglobulin is required, it is more standardized and does not require the use of subjective red cell suspension preparation required with tube testing, results are more objective (clear, eye and analyser readable) and does not require use of microscopes which is a requirement in tube testing, it is easy to automate unlike conventional tube method and thus very useful for high throughput laboratories. It also facilitates large batch testing with no risk of possible mix-up of patient sample common with tube testing and it is more sensitive and specific and has the capacity to detect very weak reactions unlike conventional tube method.
